# Crises of Amoebic Dysentery in a Carrier, Precipitated by Antityphoid Inoculations

**Published:** 1917-04

**Authors:** 


**Crises of Amoebic Dysentery in a Carrier, Precipitated by Antityphoid Inoculations.** Aide-Major Dr. Bouyer of Cauterets, Jour. de Med. de Bordeaux, Feb., reports a case having two such crises at an interval of 4 years. At both times, great prostration and severe diarrhoea occurred.

